# Multipotent Mesenchymal Stromal Cells Interact and Support Islet of Langerhans Viability and Function

**DOI:** 10.3389/fendo.2022.822191

**Published:** 2022-02-09

**Authors:** Naomi Koehler, Leo Buhler, Bernhard Egger, Carmen Gonelle-Gispert

**Affiliations:** ^1^Surgical Research Unit, Faculty of Science and Medicine, University of Fribourg, Fribourg, Switzerland; ^2^Department of Surgery, Cantonal Hospital Fribourg, Fribourg, Switzerland

**Keywords:** MSC, neonatal porcine islets, diabetes, cell encapsulation, xenotransplantion

## Abstract

Type 1 diabetes (T1D) is a widespread disease, affecting approximately 41.5 million people worldwide. It is generally treated with exogenous insulin, maintaining physiological blood glucose levels but also leading to long-term therapeutic complications. Pancreatic islet cell transplantation offers a potential alternative treatment to insulin injections. Shortage of human organ donors has raised the interest for porcine islet xenotransplantation. Neonatal porcine islets are highly available, can proliferate and mature *in vitro* as well as after transplantation *in vivo*. Despite promising preclinical results, delayed insulin secretion caused by immaturity and immunogenicity of the neonatal porcine islets remains a challenge for their clinical application. Multipotent mesenchymal stromal cells (MSCs) are known to have pro-angiogenic, anti-inflammatory and immunomodulatory effects. The current state of research emphasizes the great potential of co-culture and co-transplantation of islet cells with MSCs. Studies have shown enhanced islet proliferation and maturation, insulin secretion and graft survival, resulting in an improved graft outcome. This review summarizes the immunomodulatory and anti-inflammatory properties of MSC in the context of islet transplantation.

## Introduction

Human pancreatic islet transplantation through portal vein infusion, is a current clinical beta-cell replacement therapy to treat patients with advanced Type I Diabetes (T1D). However, live-long immunosuppression, difficulties to achieve long-term islet graft function and insulin independence as well as the shortage of suitable pancreata from heart-beating brain-dead donors for islet isolation, are still important limitations for ongoing allo-transplantation programs.

Pig islet xenotransplantation is a promising alternative to overcome the bottleneck of islet availability for the treatment of T1D. However, clinical application of pig to human islet transplantation will depend on genetic engineering of pigs to overcome immune barriers and to reduce risks of pathogen infection of porcine viruses (1). Recently, significant progress has been achieved with the transplantation of pig organs presenting several genomic modifications to prevent hyperacute rejection (2, 3) and cellular immune responses (4, 5). In immunosuppressed nonhuman primates, long-term control of diabetes by the transplantation of adult porcine islets had been successfully achieved (6, 7). Other strategies to protect porcine islets from the host immune system include islet encapsulation in semi-permeable hydrogel, such as alginate (8) functionalized by bioactive ligands or by poly(ethylene glycol) (PEG) derivatives (9, 10). Mesenchymal stem cells (MSCs) are multipotent cells and play an important role in tissue repair, angiogenesis and their immunomodulatory action on immune cells have been widely studied (11, 12). In the field of islet transplantation MSC are investigated for the improvement of islet function and graft survival after transplantation. Numerous studies of co-culture and co-transplantation with MSCs indicate a functional support. However, due to variable transplantation settings and origins of MSCs the immunomodulatory role, as well as their ability to reduce inflammatory processes *in vivo* remains controversial. This review summarizes the immunomodulatory and anti-inflammatory properties of MSC in the context of islet transplantation and evokes some of the current challenges of islet xenotransplantation.

## Multipotent Mesenchymal Stromal Cells (MSCs), Also Called Mesenchymal Stem Cells

Multipotent mesenchymal stromal cells (MSCs) are self-renewing multipotential progenitor cells, differentiating along the osteogenic, chondrogenic and adipogenic lineages (13). MSCs have first been isolated from the bone marrow over 50 years ago and bone marrow-derived MSCs (BM-MSCs) still represent the most conventional source. A variety of other tissues also contain MSCs, including adipose tissue, umbilical cord blood, Wharton’s jelly, amniotic fluid, endometrium, skin and skeletal muscle (14–22). It remains unknown which source is most suitable for the clinical use in the context of islet cell transplantation and further research is needed concerning this matter.

To facilitate isolation and expansion as well as to standardize characterization, the International Society for Cellular Therapy (ISCT) proposed three minimal criteria for defining mesenchymal stem cells. First, MSCs must be plastic-adherent when maintained in standard culture conditions. Second, MSCs must express CD105, CD73 and CD90, and lack expression of CD45, CD34, CD14 or CD11b, CD79alpha or CD19 and HLA-DR surface molecules. Third, MSCs must differentiate to osteoblasts, adipocytes and chondroblasts *in vitro* (23). MSCs have been shown to perform various beneficial functions, making them highly interesting for application in cell-based therapy, especially also for islet transplantation.

## MSCs Sustain Angiogenesis

One major limitation of islet graft survival is a delayed revascularization after transplantation. After isolation, islet cells are cut off from their oxygenation *via* micro-vascularization and are temporarily dependent after transplantation on diffusion of nutrients and oxygen in order to ensure survival. Neovascularization is finalized after approximately two weeks, however with a lower capillary density and a significantly reduced perfusion compared to islets prior to transplantation (24). Further remodeling takes up to another three months (16).

Several studies have shown a pro-angiogenic potential of MSCs. MSCs promote angiogenesis through expression and release of different pro-angiogenic cytokines, including vascular endothelial growth factor (VEGF), fibroblast growth factor (FGF), transforming growth factor beta (TGF-β), as well as annexin 1 (ANXA1), matrix metalloproteinase (MMP) and Angiopoietin-1 (Ang-1) (16). The impact of VEGF is contentious, showing not only beneficial proangiogenic but also damaging proinflammatory effects (16). MSCs seem to keep a balance through its valuable anti-inflammatory property, discussed later. Kinnaird et al. demonstrated further that co-culturing islets with MSCs enhanced neovascularization of islets through promotion of proliferation and migration of endothelial and smooth muscle cells (25).

Also, other studies showed that co-transplantation of islets with MSCs improves graft survival and function by increased neovascularization, shortening the post-transplantation ischemia period (26–28).

## Immunomodulatory Properties of MSCs

MSCs do not express co-stimulatory molecules that activate the immune system, such as CD40, CD80 or CD86 (29). Originally, it was thought that MSCs express only low or no human leukocyte antigen (HLA) class I and II molecules. It has since then been demonstrated that MSCs, like all somatic tissues, express MHC class I molecules constitutively and have the ability to express MHC class II when exposed to inflammatory cues such as interferon-γ (30). *In vitro* studies showed that, attracted by a number of complement proteins, growth factors, proinflammatory cytokines and chemokines, MSCs migrate towards sites of inflammation supporting the hypothesis that MSCs possess anti-inflammatory properties (17, 31, 32). MSCs can express potent inhibitory molecules of both, innate and adaptive immune effectors (33), however, after transplantation, this may not allow to circumvent acquired alloimmunization, as observed in human trials (30). Nevertheless, immediate events such as acute toxicity associated with the administration of MSCs have not been described (34, 35).

MSCs have also shown to exert an immunomodulatory effect through phenotype-alteration of different immune cells, including dendritic cells (DC), T- and B-cells, as well as natural killer cells (NK cells) (36). Several authors have described an inhibitory effect of MSCs on immune cell proliferation, generating an immunosuppressive local milieu (16, 37–40). Research has further shown that MSCs induce modifications of the adaptive immune system, notably T-cells, entailing T-cell anergy. MSCs act on T-cells through physically hindering contact with antigen-presenting cells (APCs) (41) or by an indirect suppression of T-cell activation *via* MSCs by hindering the maturation of DCs through cell-to-cell contact. These semi-mature DCs possess a tolerogenic phenotype, thus restraining T-cell activation (42). Also, MSCs are able to inhibit T-cell reactivity through the downregulation of proinflammatory cytokines (37, 43) and to escape cytotoxic T-cell-mediated apoptosis (44, 45). Importantly, MSCs inhibit T-lymphocyte proliferation through soluble factors, such as TGF-β1 and HGF (41, 46) or nitric oxide (47). TGF-β1 plays a well-documented role in MSCs immunomodulation, including a role in regulatory T cell (Treg) induction and/or expansion (48–50). MSCs promote the expression of regulatory T-cells (Treg) (43, 51). Early studies showed that stable islet allograft function in cynomolgus monkey was associated with increased numbers of regulatory T-cells in peripheral blood (43). Further, when co-transplanted with allogeneic islets in diabetic cynomolgus monkeys, MSCs derived from islet recipient were more efficient to prolong islet survival, when compared with 3rd party MSCs or islet derived MSCs from the donor. Using recipient-derived MSCs, they observed decreased number of memory T cells, reduced anti-donor T cell proliferation and higher Treg:T cell ratios (52).

## Co-Transplantation of Islets With MSCs From Different Sources

Various possible sources of MSCs have been tested for co-transplantation with islet cells so far.

In murine models, several studies showed improved and prolonged graft survival, function, morphology and revascularization, as well as induction of beta cell proliferation following transplantation of murine islets with autologous (53), syngeneic (27, 54, 55), allogeneic (27, 56–59) or xenogeneic MSCs (60). In mice, co-transplantation of autologous MSCs delayed islet allograft rejection and generated a local immune-privileged site in mice (53). Rackham et al. studied the effects of co-transplantation of syngeneic murine MSCs and islet cells, and observed an improved graft outcome (54). In a subsequent study they examined the underlying factors, suggesting Annexin A1 to be a key contributor to the improved graft function through direct and indirect mechanisms (61). The exact mechanisms remain unclear.

Co-transplantation of islets with MSCs in syngeneic rodent models showed better outcomes of islet survival and function than islets transplanted alone (26, 27, 62). Karaoz et al. described an improved islet function after co-culturing allogenic rat MSCs and islet cells, suggesting paracrine actions through IL-6, TGF-β1, osteopontin and fibronectin (59). Further, allogeneic MSCs resulted in improved islet xenograft survival and function in immune-competent diabetic mice (63). In cynomolgus monkeys, intraportal co-infusion of allogenic MSCs and islets, increased islet engraftment and function, shown by a reduced number of islets necessary to reach normoglycemia (43).

Co-encapsulation studies using islets versus islets and MSC also showed beneficial effects on islet function (64–66). Intraperitoneally syngeneic transplantation of co-encapsulated islets and MSCs showed significantly lower glycaemia compared to islets encapsulated alone. By week 6, 71% of mice transplanted with islets and MSCs were cured, whereas only 16% of the islets-alone group was cured at that point. Interestingly, islet area in recovered capsules was significantly higher when co-encapsulated with MSCs suggesting that MSCs promote survival of islet cells independently from its effects on revascularization. In this study co-encapsulation of islets with MSC did not inhibit pericapsular fibrotic overgrowth (PFO), suggesting that MSCs have no influence on the inflammatory process that causes fibrotic overgrowth (64). PFO is an inflammatory host reaction, induced through the leakage of antigens from semi-permeable microcapsules, that severely impairs islet viability and graft function. However, in a mouse model of islet allotransplantation, co-encapsulation of MSCs (stimulated or not with a cocktail of pro-inflammatory cytokines) with islets in alginate microcapsules, prevented pericapsular fibrotic overgrowth (PFO) compared to islets encapsulated alone (66). Further mice receiving islets co-encapsulated with stimulated and unstimulated MSC achieved higher percentages of normoglycemic mice (100% versus 71.4%, respectively) compared to mice transplanted with islets encapsulated alone (9.1%). Similarly, *in vitro* rat MSCs and rat islet cells when co-encapsulated in a ligand-functionalized polyethylene glycol (PEG) hydrogel (67) led to a doubling of the stimulation index compared to islets encapsulated alone. Co-encapsulation of islet cells and MSCs in addition with cell adhesion peptides led to a significant sevenfold increase of the stimulation index compared to islets encapsulated alone (67).

Human islets co-cultured in direct cell contact with human MSCs compared to islets co-cultured with MSCs but without cell-to-cell contact, displayed significantly enhanced insulin secretion in the presence of cell-to-cell contact. This effect was identified to be dependent on N-cadherin interaction, since impeding N-cadherin interaction with antibodies led to a reversal of the enhanced insulin secretion. Additionally, mice transplanted intraperitoneally with human islets co-encapsulated with MSCs in hydrogel microspheres, composed of calcium alginate and covalently crosslinked to polyethylene glycol showed significantly lower blood glucose levels and prolonged islet graft survival (57). Others have shown that improved graft function correlates with enhanced revascularization of islets transplanted under the kidney capsule (68–70). Accordingly, research findings revealed significantly higher apoptosis rates in islet cells cultured without MSCs (16).

Taken altogether, these findings support the hypothesis that co-transplantation of MSCs and islet cells is beneficial and that MSC**s** are useful for future therapeutic applications.

## Improved Neonatal Porcine Islet Function, Survival and Graft Outcome

**Figure 1:**
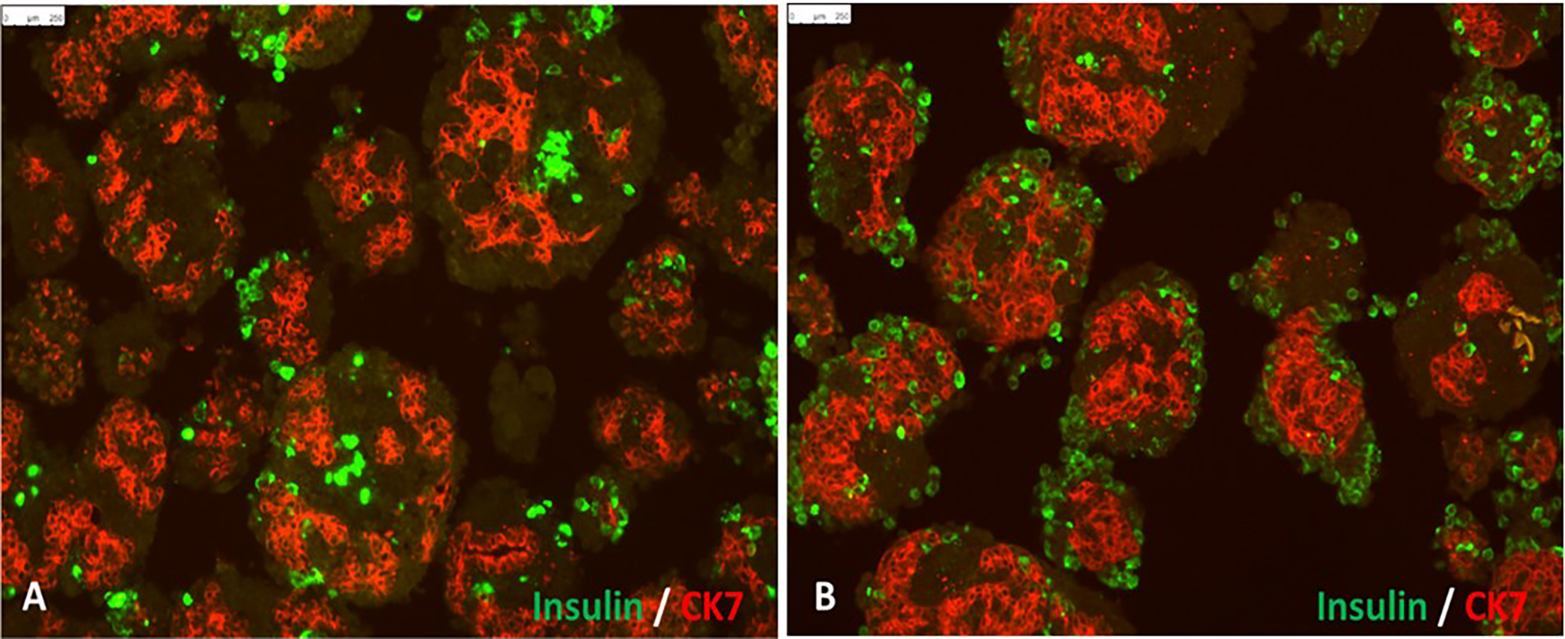
*In vitro* differentiation of isolated porcine pancreatic islet cell clusters: Panel **(A)** shows cell clusters containing insulin-positive beta cells (green) and CK7-positive pancreatic exocrine tissue (red) at 3 days after isolation. Panel **(B)** shows pancreatic islet cell clusters 7 days after culture in neonatal pig islet differentiation media (scale bar represents 250 μm).

The main disadvantage of neonatal or juvenile porcine islets, also called porcine pancreatic islet cell clusters (ICCs), is their lack of integrity and maturity. ICCs are obtained by *in vitro* digestion of neonatal or juvenile pig pancreas with subsequent short time culture in a specific maturation media to increase the beta cell mass for transplantation (**Figure 1**) (71). Also, porcine pancreatic ICC co-transplanted with human MSC into immune deficient diabetic mice reached normoglycemia significantly earlier than mice transplanted with ICC alone (60).

He et al. demonstrated an improved and accelerated development of ICCs in diabetic rhesus monkeys after co-transplantation with allogeneic simian MSCs into diabetic rhesus monkeys (72). Additionally, the group described an enhanced expression of genes implicated in the development of endocrine cells and insulin and further demonstrated enhanced expression and activation of PDGFR-α in neonatal islets through MSCs confirming earlier studies demonstrating the capability of PDGFR-α to stimulate beta-cell proliferation (73). Further, He et al. suggest an inhibition of the Notch1 signaling provoked by PDGFR-α, leading to an improved islet development and maturation. It is known that Notch1 downregulates the expression of several genes and transcription factors implicated in the development of endocrine cells and insulin (72, 74). Juvenile porcine exocrine pancreas-derived MSCs (pMSCs) co-cultured with direct cell to cell contact of juvenile porcine ICCs significantly enhanced beta-cell function, suggesting that cell signaling *via* adhesion molecules are important (57, 65). However, co-encapsulation of such ICCs with pMSCs do not effectively prevent PFO and graft survival was rapidly impaired after transplantation of capsules in immunocompetent mice. Therefore, further research is required to enable efficient long-term survival of encapsulated juvenile porcine islets. Possible approaches being evaluation of modified alginate chemical composition (75) or the use of different anti-fibrotic polymers (65).

## Immunomodulation Strategies to Increase Xenograft Survival

To overcome the immunological barrier between pig and humans, genetic modifications have been performed in pig strains to reduce immunogenicity of organs and tissue. The first genetically modified pig, i.e. with a single human transgene for a complement regulating gene (hDAF), allowed survival of pig organs in immunosuppressed non-human primates for several months. Since then, genetic engineering, using CRISPR-CAS9, allowed cloning of animals with additional genetic modifications. Today, pigs with over 10 genetic modifications, both, deletions of pig antigens and inclusions of human transgenes are under investigation for transplantation purposes (76). Immunosuppressive regimens are still necessary but recently heart transplantation from genetically modified pigs [α1,3-galactosyltransferase-knockout and knock in human CD46 (77, 78) and thrombomodulin (79)] to a non-human primate (baboon) reached long term survival of 195 days (80).

Furthermore, immunoregulatory therapies (tolerance induction) using Treg-based therapeutic approaches are under investigation. Regulatory T cells (Tregs) are immune-suppressive T cells that are critical for the maintenance of tolerance *in vivo* (81). Chimeric antigen receptors (CARs) are synthetic fusion proteins that have been developed to genetically modify T cells in order to create a specificity toward designated antigens. The application of the CAR technology to Tregs, may allow to reduce immune responses for solid organ and cell transplantation. CAR Treg therapies are currently developed using genetic modifications for xenogenic pig antigens with the aim to improve graft acceptance of xenotransplanted tissue i.e. porcine islets. This might be achieved through infusion of *ex vivo* expansion of donor-specific Tregs (55, 82).

CAR-Tregs technology started with a study of MacDonald and colleagues which successfully transduced human Tregs with a CAR targeting the human leukocyte antigen (HLA) class I-A2 (A2-CAR) (83). In a human skin xenograft transplant model, HLA class I-A2 specific CAR-Tregs alleviated rejection of skin transplants (84). Since co-transplantation of autologous MSC delayed islet allograft rejection, it is possible that genetically modified, MSC, could be exploited as a target cell in porcine ICC xenografts to foster islet function and to increase trafficking and activation of adoptive transferred CAR-Treg cells to increase tolerance toward pig ICC xenografts.

An additional challenge for islet transplantation is the precise quantification of beta cell mass (BCM) or endocrine cell mass (ECM) *in vivo*. Imaging the progressive loss of beta cells following islet transplantation should allow the development of individualized therapies for the management of patients post-transplant (85). Recently, a suitable biomarker for beta cell quantification, the dipeptidyl aminopeptidase-like protein 6 (DPP6) has been identified as a promising target for human BCM imaging in healthy individuals as well as diabetic patients (86, 87). First imaging and biodistribution studies using SPECT/CT and radiolabeled high-affinity camelid single-domain antibody (nanobody) directed specifically against human DPP6, allowed to visualize transplanted DPP6-expressing Kelly neuroblastoma cells or insulin-producing human EndoC-βH1 cells in immunodeficient mice. Importantly, neonatal pig islets expressing near-infrared fluorescent protein (iRFP) were non-invasively monitored through multispectral optoacoustic tomography (MSOT). MSOT signals, obtained after islet transplantation under the kidney capsule in mice, and obtained after subcutaneous and intramuscular islet transplantation in pigs, allowed to distinguish graft mass changes (88). Such reporter gene-expressing islets are also promising tools to evaluate the efficacy of newly developed biomaterials for encapsulation and transplantation of porcine islets.

## Conclusion

Diabetes is a worldwide disease, affecting over 40 million people and putting an important burden on the healthcare system. Exogenous insulin represents the predominant treatment modality for type 1 diabetes, but is associated with long-term complications. Islet cell transplantation is a highly promising approach for treating type 1 diabetes aiming at reestablishing a physiological insulin secretion through replacement of the endocrine tissue. Despite improving preclinical and clinical results over the past decades, the need for immunosuppression and donor shortage limits the clinical application of this procedure. The implementation of porcine pancreatic ICCs with porcine MSC might represent a promising alternative to help to overcome the problem of donor shortage; especially neonatal or juvenile pigs providing high islet yields. Encapsulation techniques could resolve the need for immunosuppression, shielding the islets from immune attacks while still enabling the exchange of oxygen, insulin and nutrients. Yet, delayed and impaired graft outcome due to immature islet cells and the formation of pericapsular fibrosis continue to severely limit the clinical application of encapsulated islet transplantation.

## Author Contributions

NK: designed and wrote the manuscript; LB contributed to manuscript writing; LB and BE: critically revised the manuscript; CGG: designed, contributed to manuscript writing; supervised the writing of the manuscript. All authors contributed to the article and approved the submitted version.

## Conflict of Interest

The authors declare that the research was conducted in the absence of any commercial or financial relationships that could be construed as a potential conflict of interest.

## Publisher’s Note

All claims expressed in this article are solely those of the authors and do not necessarily represent those of their affiliated organizations, or those of the publisher, the editors and the reviewers. Any product that may be evaluated in this article, or claim that may be made by its manufacturer, is not guaranteed or endorsed by the publisher.

## References

[1] Denner J. Porcine Lymphotropic Herpesviruses (Plhvs) and Xenotranplantation. Viruses (2021) 13(6):1072. doi: 10.3390/v13061072 34199939 PMC8229715

[2] Lai L Kolber-Simonds D Park KW Cheong HT Greenstein JL Im GS. Production of Alpha-1,3-Galactosyltransferase Knockout Pigs by Nuclear Transfer Cloning. Science (2002) 295(5557):1089–92. doi: 10.1126/science.1068228 11778012

[3] Phelps CJ Koike C Vaught TD Boone J Wells KD Chen SH. Production of Alpha 1,3-Galactosyltransferase-Deficient Pigs. Science (2003) 299(5605):411–4. doi: 10.1126/science.1078942 PMC3154759 12493821

[4] Liu Z Hu W He T Dai Y Hara H Bottino R. Pig-to-Primate Islet Xenotransplantation: Past, Present, and Future. Cell Transplant (2017) 26(6):925–47. doi: 10.3727/096368917X694859 PMC5657750 28155815

[5] Hryhorowicz M Lipinski D Hryhorowicz S Nowak-Terpilowska A Ryczek N Zeyland J. Application of Genetically Engineered Pigs in Biomedical Research. Genes (Basel) (2020) 11(6):670. doi: 10.3390/genes11060670 PMC7349405 32575461

[6] Shin JS Kim JM Kim JS Min BH Kim YH Kim HJ. Long-Term Control of Diabetes in Immunosuppressed Nonhuman Primates (NHP) by the Transplantation of Adult Porcine Islets. Am J Transplant (2015) 15(11):2837–50. doi: 10.1111/ajt.13345 26096041

[7] Shin JS Kim JM Min BH Yoon IH Kim HJ Kim JS. Pre-Clinical Results in Pig-to-Non-Human Primate Islet Xenotransplantation Using Anti-CD40 Antibody (2C10R4)-Based Immunosuppression. Xenotransplantation (2018) 25(1):e12356. doi: 10.1111/xen.12356 PMC5809197 29057561

[8] Ghasemi A Akbari E Imani R. An Overview of Engineered Hydrogel-Based Biomaterials for Improved Beta-Cell Survival and Insulin Secretion. Front Bioeng Biotechnol (2021) 9:662084. doi: 10.3389/fbioe.2021.662084 34513805 PMC8427138

[9] Laporte C Tubbs E Pierron M Gallego A Moisan A Lamarche F. Improved Human Islets’ Viability and Functionality With Mesenchymal Stem Cells and Arg-Gly-Asp Tripeptides Supplementation of Alginate Micro-Encapsulated Islets In Vitro. Biochem Biophys Res Commun (2020) 528(4):650–7. doi: 10.1016/j.bbrc.2020.05.107 32513541

[10] Passemard S Szabo L Noverraz F Montanari E Gonelle-Gispert C Buhler LH. Synthesis Strategies to Extend the Variety of Alginate-Based Hybrid Hydrogels for Cell Microencapsulation. Biomacromolecules (2017) 18(9):2747–55. doi: 10.1021/acs.biomac.7b00665 28742341

[11] Shi M Liu ZW Wang FS. Immunomodulatory Properties and Therapeutic Application of Mesenchymal Stem Cells. Clin Exp Immunol (2011) 164(1):1–8. doi: 10.1111/j.1365-2249.2011.04327.x PMC3074211 21352202

[12] Castro-Manrreza ME Montesinos JJ. Immunoregulation by Mesenchymal Stem Cells: Biological Aspects and Clinical Applications. J Immunol Res (2015) 2015:394917. doi: 10.1155/2015/394917 25961059 PMC4417567

[13] Sudo K Kanno M Miharada K Ogawa S Hiroyama T Saijo K. Mesenchymal Progenitors Able to Differentiate Into Osteogenic, Chondrogenic, and/or Adipogenic Cells In Vitro Are Present in Most Primary Fibroblast-Like Cell Populations. Stem Cells (2007) 25(7):1610–7. doi: 10.1634/stemcells.2006-0504 17395773

[14] Kern S Eichler H Stoeve J Klüter H Bieback K. Comparative Analysis of Mesenchymal Stem Cells From Bone Marrow, Umbilical Cord Blood, or Adipose Tissue. Stem Cells (2006) 24(5):1294–301. doi: 10.1634/stemcells.2005-0342 16410387

[15] da Silva Meirelles L Chagastelles PC Nardi NB. Mesenchymal Stem Cells Reside in Virtually All Post-Natal Organs and Tissues. J Cell Sci (2006) 119(Pt 11):2204–13. doi: 10.1242/jcs.02932 16684817

[16] Gamble A Pawlick R Pepper AR Bruni A Adesida A Senior PA. Improved Islet Recovery and Efficacy Through Co-Culture and Co-Transplantation of Islets With Human Adipose-Derived Mesenchymal Stem Cells. PLoS One (2018) 13(11):e0206449. doi: 10.1371/journal.pone.0206449 30419033 PMC6231609

[17] Kang EJ Lee YH Kim MJ Lee YM Kumar BM Jeon BG. Transplantation of Porcine Umbilical Cord Matrix Mesenchymal Stem Cells in a Mouse Model of Parkinson’s Disease. J Tissue Eng Regener Med (2013) 7(3):169–82. doi: 10.1002/term.504 22081626

[18] Zhang W Liu XC Yang L Zhu DL Zhang YD Chen Y. Wharton’s Jelly-Derived Mesenchymal Stem Cells Promote Myocardial Regeneration and Cardiac Repair After Miniswine Acute Myocardial Infarction. Coron Artery Dis (2013) 24(7):549–58. doi: 10.1097/MCA.0b013e3283640f00 23892469

[19] Miernik K Karasinski J. Porcine Uterus Contains a Population of Mesenchymal Stem Cells. Reproduction (2012) 143(2):203–9. doi: 10.1530/REP-11-0202 22065860

[20] Subbarao RB Ullah I Kim EJ Jang SJ Lee WJ Jeon RH. Characterization and Evaluation of Neuronal Trans-Differentiation With Electrophysiological Properties of Mesenchymal Stem Cells Isolated From Porcine Endometrium. Int J Mol Sci (2015) 16(5):10934–51. doi: 10.3390/ijms160510934 PMC4463684 26006231

[21] Park BW Kang DH Kang EJ Byun JH Lee JS. Peripheral Nerve Regeneration Using Autologous Porcine Skin-Derived Mesenchymal Stem Cells. J Tissue Eng Regener Med (2012) 6(2):113–24. doi: 10.1002/term.404 21337707

[22] Kang EJ Byun JH Choi YJ Maeng GH Lee SL Kang DH. In Vitro and In Vivo Osteogenesis of Porcine Skin-Derived Mesenchymal Stem Cell-Like Cells With a Demineralized Bone and Fibrin Glue Scaffold. Tissue Eng Part A (2010) 16(3):815–27. doi: 10.1089/ten.tea.2009.0439 19778183

[23] Dominici M Le Blanc K Mueller I Slaper-Cortenbach I Marini F Krause D. Minimal Criteria for Defining Multipotent Mesenchymal Stromal Cells. The International Society for Cellular Therapy Position Statement. Cytotherapy (2006) 8(4):315–7. doi: 10.1080/14653240600855905 16923606

[24] Carlsson P-O Palm F Mattsson G. Low Revascularization of Experimentally Transplanted Human Pancreatic Islets. J Clin Endocrinol Metab (2003) 87:5418–23. doi: 10.1210/jc.2002-020728 12466329

[25] Kinnaird T Stabile E Burnett MS Lee CW Barr S Fuchs S. Marrow-Derived Stromal Cells Express Genes Encoding a Broad Spectrum of Arteriogenic Cytokines and Promote In Vitro and In Vivo Arteriogenesis Through Paracrine Mechanisms. Circ Res (2004) 94(5):678–85. doi: 10.1161/01.RES.0000118601.37875.AC 14739163

[26] Figliuzzi M Cornolti R Perico N Rota C Morigi M Remuzzi G. Bone Marrow-Derived Mesenchymal Stem Cells Improve Islet Graft Function in Diabetic Rats. Transplant Proc (2009) 41(5):1797–800. doi: 10.1016/j.transproceed.2008.11.015 19545731

[27] Ito T Itakura S Todorov I Rawson J Asari S Shintaku J. Mesenchymal Stem Cell and Islet Co-Transplantation Promotes Graft Revascularization and Function. Transplantation (2010) 89(12):1438–45. doi: 10.1097/TP.0b013e3181db09c4 20568673

[28] Päth G Perakakis N Mantzoros CS Seufert J. Stem Cells in the Treatment of Diabetes Mellitus - Focus on Mesenchymal Stem Cells. Metabolism (2019) 90:1–15. doi: 10.1016/j.metabol.2018.10.005 30342065

[29] Wang Q Ding G Xu X. Immunomodulatory Functions of Mesenchymal Stem Cells and Possible Mechanisms. Histol Histopathol (2016) 31(9):949–59. doi: 10.14670/HH-11-750 26932157

[30] Ankrum JA Ong JF Karp JM. Mesenchymal Stem Cells: Immune Evasive, Not Immune Privileged. Nat Biotechnol (2014) 32(3):252–60. doi: 10.1038/nbt.2816 PMC4320647 24561556

[31] English K. Mechanisms of Mesenchymal Stromal Cell Immunomodulation. Immunol Cell Biol (2013) 91(1):19–26. doi: 10.1038/icb.2012.56 23090487

[32] Azhar M Yin M Bommireddy R Duffy JJ Yang J Pawlowski SA. Generation of Mice With a Conditional Allele for Transforming Growth Factor Beta 1 Gene. Genesis (2009) 47(6):423–31. doi: 10.1002/dvg.20516 PMC2766615 19415629

[33] Uccelli A Moretta L Pistoia V. Mesenchymal Stem Cells in Health and Disease. Nat Rev Immunol (2008) 8(9):726–36. doi: 10.1038/nri2395 19172693

[34] Lalu MM McIntyre L Pugliese C Fergusson D Winston BW Marshall JC. Safety of Cell Therapy With Mesenchymal Stromal Cells (Safecell): A Systematic Review and Meta-Analysis of Clinical Trials. PLoS One (2012) 7(10):e47559. doi: 10.1371/journal.pone.0047559 23133515 PMC3485008

[35] Koç ON Gerson SL Cooper BW Dyhouse SM Haynesworth SE Caplan AI. Rapid Hematopoietic Recovery After Coinfusion of Autologous-Blood Stem Cells and Culture-Expanded Marrow Mesenchymal Stem Cells in Advanced Breast Cancer Patients Receiving High-Dose Chemotherapy. J Clin Oncol (2000) 18(2):307–16. doi: 10.1200/JCO.2000.18.2.307 10637244

[36] Aggarwal S Pittenger MF. Human Mesenchymal Stem Cells Modulate Allogeneic Immune Cell Responses. Blood (2005) 105(4):1815–22. doi: 10.1182/blood-2004-04-1559 15494428

[37] Glennie S Soeiro I Dyson PJ Lam EW-F Dazzi F. Bone Marrow Mesenchymal Stem Cells Induce Division Arrest Anergy of Activated T Cells. Blood (2005) 105(7):2821–7. doi: 10.1182/blood-2004-09-3696 15591115

[38] Corcione A Benvenuto F Ferretti E Giunti D Cappiello V Cazzanti F. Human Mesenchymal Stem Cells Modulate B-Cell Functions. Blood (2006) 107(1):367–72. doi: 10.1182/blood-2005-07-2657 16141348

[39] Spaggiari GM Capobianco A Becchetti S Mingari MC Moretta L. Mesenchymal Stem Cell-Natural Killer Cell Interactions: Evidence That Activated NK Cells Are Capable of Killing Mscs, Whereas Mscs can Inhibit IL-2-Induced NK-Cell Proliferation. Blood (2006) 107(4):1484–90. doi: 10.1182/blood-2005-07-2775 16239427

[40] Bartholomew A Sturgeon C Siatskas M Ferrer K McIntosh K Patil S. Mesenchymal Stem Cells Suppress Lymphocyte Proliferation In Vitro and Prolong Skin Graft Survival In Vivo. Exp Hematol (2002) 30(1):42–8. doi: 10.1016/S0301-472X(01)00769-X 11823036

[41] Krampera M Glennie S Dyson J Scott D Laylor R Simpson E. Bone Marrow Mesenchymal Stem Cells Inhibit the Response of Naive and Memory Antigen-Specific T Cells to Their Cognate Peptide. Blood (2003) 101(9):3722–9. doi: 10.1182/blood-2002-07-2104 12506037

[42] Beyth S Borovsky Z Mevorach D Liebergall M Gazit Z Aslan H. Human Mesenchymal Stem Cells Alter Antigen-Presenting Cell Maturation and Induce T-Cell Unresponsiveness. Blood (2005) 105(5):2214–9. doi: 10.1182/blood-2004-07-2921 15514012

[43] Berman DM Willman MA Han D Kleiner G Kenyon NM Cabrera O. Mesenchymal Stem Cells Enhance Allogeneic Islet Engraftment in Nonhuman Primates. Diabetes (2010) 59(10):2558–68. doi: 10.2337/db10-0136 PMC3279532 20622174

[44] Ryan JM Barry FP Murphy JM Mahon BP. Mesenchymal Stem Cells Avoid Allogeneic Rejection. J Inflamm (London England) (2005) 2:8–8. doi: 10.1186/1476-9255-2-8 PMC1215510 16045800

[45] Rasmusson I Uhlin M Le Blanc K Levitsky V. Mesenchymal Stem Cells Fail to Trigger Effector Functions of Cytotoxic T Lymphocytes. J Leukocyte Biol (2007) 82(4):887–93. doi: 10.1189/jlb.0307140 17609339

[46] Di Nicola M Carlo-Stella C Magni M Milanesi M Longoni PD Matteucci P. Human Bone Marrow Stromal Cells Suppress T-Lymphocyte Proliferation Induced by Cellular or Nonspecific Mitogenic Stimuli. Blood (2002) 99(10):3838–43. doi: 10.1182/blood.V99.10.3838 11986244

[47] Sato K Ozaki K Oh I Meguro A Hatanaka K Nagai T. Nitric Oxide Plays a Critical Role in Suppression of T-Cell Proliferation by Mesenchymal Stem Cells. Blood (2007) 109(1):228–34. doi: 10.1182/blood-2006-02-002246 16985180

[48] Carrillo-Galvez AB Cobo M Cuevas-Ocana S Gutierrez-Guerrero A Sanchez-Gilabert A Bongarzone P. Mesenchymal Stromal Cells Express GARP/LRRC32 on Their Surface: Effects on Their Biology and Immunomodulatory Capacity. Stem Cells (2015) 33(1):183–95. doi: 10.1002/stem.1821 PMC4309416 25182959

[49] Patel SA Meyer JR Greco SJ Corcoran KE Bryan M Rameshwar P. Mesenchymal Stem Cells Protect Breast Cancer Cells Through Regulatory T Cells: Role of Mesenchymal Stem Cell-Derived TGF-Beta. J Immunol (2010) 184(10):5885–94. doi: 10.4049/jimmunol.0903143 20382885

[50] Salazar KD Lankford SM Brody AR. Mesenchymal Stem Cells Produce Wnt Isoforms and TGF-Beta1 That Mediate Proliferation and Procollagen Expression by Lung Fibroblasts. Am J Physiol Lung Cell Mol Physiol (2009) 297(5):L1002–11. doi: 10.1152/ajplung.90347.2008 PMC2777498 19734317

[51] De Miguel MP Fuentes-Julián S Blázquez-Martínez A Pascual CY Aller MA Arias J. Immunosuppressive Properties of Mesenchymal Stem Cells: Advances and Applications. Curr Mol Med (2012) 12(5):574–91. doi: 10.2174/156652412800619950 22515979

[52] Kenyon NS Willman MA Han D Leeman RS Rabassa A Diaz WL. Extended Survival vs Accelerated Rejection of Nonhuman Primate Islet Allografts: Effect of Mesenchymal Stem Cell Source and Timing. Am J Transplant (2021) 21(11):3524–37. doi: 10.1111/ajt.16693 PMC9034438 34008325

[53] Ben Nasr M Vergani A Avruch J Liu L Kefaloyianni E D'Addio F. Co-Transplantation of Autologous Mscs Delays Islet Allograft Rejection and Generates a Local Immunoprivileged Site. Acta Diabetol (2015) 52(5):917–27. doi: 10.1007/s00592-015-0735-y PMC4968999 25808641

[54] Rackham CL Chagastelles PC Nardi NB Hauge-Evans AC Jones PM King AJF. Co-Transplantation of Mesenchymal Stem Cells Maintains Islet Organisation and Morphology in Mice. Diabetologia (2011) 54(5):1127–35. doi: 10.1007/s00125-011-2053-4 21267536

[55] Huang D Wang Y Hawthorne WJ Hu M Hawkes J Burns H. Ex Vivo-Expanded Baboon CD39 + Regulatory T Cells Prevent Rejection of Porcine Islet Xenografts in NOD-SCID IL-2rgamma(-/-) Mice Reconstituted With Baboon Peripheral Blood Mononuclear Cells. Xenotransplantation (2017) 24(6):e12344. doi: 10.1111/xen.12344 28963731

[56] Longoni B Szilagyi E Quaranta P Paoli GT Tripodi S Urbani S. Mesenchymal Stem Cells Prevent Acute Rejection and Prolong Graft Function in Pancreatic Islet Transplantation. Diabetes Technol Ther (2010) 12(6):435–46. doi: 10.1089/dia.2009.0154 20470228

[57] Montanari E Meier RPH Mahou R Seebach JD Wandrey C Gerber-Lemaire S. Multipotent Mesenchymal Stromal Cells Enhance Insulin Secretion From Human Islets via N-Cadherin Interaction and Prolong Function of Transplanted Encapsulated Islets in Mice. Stem Cell Res Ther (2017) 8(1):199. doi: 10.1186/s13287-017-0646-7 28962589 PMC5622460

[58] Gao X Song L Shen K Wang H Qian M Niu W. Bone Marrow Mesenchymal Stem Cells Promote the Repair of Islets From Diabetic Mice Through Paracrine Actions. Mol Cell Endocrinol (2014) 388(1):41–50. doi: 10.1016/j.mce.2014.03.004 24667703

[59] Karaoz E Genç ZS Demircan PÇ Aksoy A Duruksu G. Protection of Rat Pancreatic Islet Function and Viability by Coculture With Rat Bone Marrow-Derived Mesenchymal Stem Cells. Cell Death Dis (2010) 1(4):e36–6. doi: 10.1038/cddis.2010.14 PMC3032304 21364643

[60] Hayward JA Ellis CE Seeberger K Lee T Salama B Mulet-Sierra A. Cotransplantation of Mesenchymal Stem Cells With Neonatal Porcine Islets Improve Graft Function in Diabetic Mice. Diabetes (2017) 66(5):1312–21. doi: 10.2337/db16-1068 28246290

[61] Rackham CL Vargas AE Hawkes RG Amisten S Persaud SJ Austin ALF. Annexin A1 Is a Key Modulator of Mesenchymal Stromal Cell–Mediated Improvements in Islet Function. Diabetes (2016) 65(1):129–39. doi: 10.2337/db15-0990 26470781

[62] Borg DJ Weigelt M Wilhelm C Gerlach M Bickle M Speier S. Mesenchymal Stromal Cells Improve Transplanted Islet Survival and Islet Function in a Syngeneic Mouse Model. Diabetologia (2014) 57(3):522–31. doi: 10.1007/s00125-013-3109-4 24253203

[63] Perez-Basterrechea M Obaya AJ Meana A Otero J Esteban MM. Cooperation by Fibroblasts and Bone Marrow-Mesenchymal Stem Cells to Improve Pancreatic Rat-to-Mouse Islet Xenotransplantation. PloS One (2013) 8(8):e73526. doi: 10.1371/journal.pone.0073526 24009755 PMC3756982

[64] Kerby A Jones ES Jones PM King AJ. Co-Transplantation of Islets With Mesenchymal Stem Cells in Microcapsules Demonstrates Graft Outcome can be Improved in an Isolated-Graft Model of Islet Transplantation in Mice. Cytotherapy (2013) 15(2):192–200. doi: 10.1016/j.jcyt.2012.10.018 23321331

[65] Montanari E Szabo L Balaphas A Meyer J Perriraz-Mayer N Pimenta J. Multipotent Mesenchymal Stromal Cells Derived From Porcine Exocrine Pancreas Improve Insulin Secretion From Juvenile Porcine Islet Cell Clusters. Xenotransplantation (2021) 28(3):e12666. doi: 10.1111/xen.12666 33538027

[66] Vaithilingam V Evans MDM Lewy DM Bean PA Bal S Tuch BE. Co-Encapsulation and Co-Transplantation of Mesenchymal Stem Cells Reduces Pericapsular Fibrosis and Improves Encapsulated Islet Survival and Function When Allografted. Sci Rep (2017) 7(1):10059. doi: 10.1038/s41598-017-10359-1 28855611 PMC5577272

[67] Bal T Nazli C Okcu A Duruksu G Karaöz E Kizilel S. Mesenchymal Stem Cells and Ligand Incorporation in Biomimetic Poly(Ethylene Glycol) Hydrogels Significantly Improve Insulin Secretion From Pancreatic Islets. J Tissue Eng Regenerative Med (2017) 11(3):694–703. doi: 10.1002/term.1965 25393526

[68] Bertera S Knoll MF Knoll C Hara H Kimbrel EA Kouris NA. Human Hemangioblast-Derived Mesenchymal Stem Cells Promote Islet Engraftment in a Minimal Islet Mass Transplantation Model in Mice. Front Med (Lausanne) (2021) 8:660877. doi: 10.3389/fmed.2021.660877 33937296 PMC8081894

[69] Cunha JP Leuckx G Sterkendries P Korf H Bomfim-Ferreira G Overbergh L. Human Multipotent Adult Progenitor Cells Enhance Islet Function and Revascularisation When Co-Transplanted as a Composite Pellet in a Mouse Model of Diabetes. Diabetologia (2017) 60(1):134–42. doi: 10.1007/s00125-016-4120-3 PMC6518081 27704164

[70] Selmani Z Naji A Zidi I Favier B Gaiffe E Obert L. Human Leukocyte Antigen-G5 Secretion by Human Mesenchymal Stem Cells Is Required to Suppress T Lymphocyte and Natural Killer Function and to Induce CD4+CD25highFOXP3+ Regulatory T Cells. Stem Cells (2008) 26(1):212–22. doi: 10.1634/stemcells.2007-0554 17932417

[71] Korbutt GS Elliott JF Ao Z Smith DK Warnock GL Rajotte RV. Large Scale Isolation, Growth, and Function of Porcine Neonatal Islet Cells. J Clin Invest (1996) 97(9):2119–29. doi: 10.1172/JCI118649 PMC507287 8621802

[72] He S Wang C Du X Chen Y Zhao J Tian B. Mscs Promote the Development and Improve the Function of Neonatal Porcine Islet Grafts. FASEB J (2018) 32(6):3242–53. doi: 10.1096/fj.201700991R 29401607

[73] Chen H Gu X Liu Y Wang J Wirt SE Bottino R. PDGF Signalling Controls Age-Dependent Proliferation in Pancreatic β-Cells. Nature (2011) 478(7369):349–55. doi: 10.1038/nature10502 PMC3503246 21993628

[74] Hald J Hjorth JP German MS Madsen OD Serup P Jensen J. Activated Notch1 Prevents Differentiation of Pancreatic Acinar Cells and Attenuate Endocrine Development. Dev Biol (2003) 260(2):426–37. doi: 10.1016/S0012-1606(03)00326-9 12921743

[75] Vaithilingam V Bal S Tuch BE. Encapsulated Islet Transplantation: Where do We Stand? Rev Diabetes Stud (2017) 14(1):51–78. doi: 10.1900/RDS.2017.14.51 PMC6115002 28632821

[76] Meier RPH Muller YD Balaphas A Morel P Pascual M Seebach JD. Xenotransplantation: Back to the Future? Transpl Int (2018) 31(5):465–77. doi: 10.1111/tri.13104 29210109

[77] Cooper DKC Hara H Iwase H Yamamoto T Jagdale A Kumar V. Clinical Pig Kidney Xenotransplantation: How Close Are We? J Am Soc Nephrol (2020) 31(1):12–21. doi: 10.1681/ASN.2019070651 31792154 PMC6934994

[78] Jagdale A Nguyen H Li J Burnette K Ayares D Cooper DKC. Does Expression of a Human Complement-Regulatory Protein on Xenograft Cells Protect Them From Systemic Complement Activation? Int J Surg (2020) 83:184–8. doi: 10.1016/j.ijsu.2020.09.034 PMC7686296 32987208

[79] Hara H Iwase H Nguyen H Miyagawa Y Kuravi K Foote JB. Stable Expression of the Human Thrombomodulin Transgene in Pig Endothelial Cells Is Associated With a Reduction in the Inflammatory Response. Cytokine (2021) 148:155580. doi: 10.1016/j.cyto.2021.155580 34099346 PMC8511266

[80] Langin M Mayr T Reichart B Michel S Buchholz S Guethoff S. Consistent Success in Life-Supporting Porcine Cardiac Xenotransplantation. Nature (2018) 564(7736):430–3. doi: 10.1038/s41586-018-0765-z 30518863

[81] Pilat N Sprent J. Treg Therapies Revisited: Tolerance Beyond Deletion. Front Immunol (2020) 11:622810. doi: 10.3389/fimmu.2020.622810 33633742 PMC7902070

[82] Ezzelarab MB. Regulatory T Cells From Allo- to Xenotransplantation: Opportunities and Challenges. Xenotransplantation (2018) 25(3):e12415. doi: 10.1111/xen.12415 29913039

[83] MacDonald KG Hoeppli RE Huang Q Gillies J Luciani DS Orban PC. Alloantigen-Specific Regulatory T Cells Generated With a Chimeric Antigen Receptor. J Clin Invest (2016) 126(4):1413–24. doi: 10.1172/JCI82771 PMC4811124 26999600

[84] Boardman DA Philippeos C Fruhwirth GO Ibrahim MA Hannen RF Cooper D. Expression of a Chimeric Antigen Receptor Specific for Donor HLA Class I Enhances the Potency of Human Regulatory T Cells in Preventing Human Skin Transplant Rejection. Am J Transplant (2017) 17(4):931–43. doi: 10.1111/ajt.14185 28027623

[85] Demine S Schulte ML Territo PR Eizirik DL. Beta Cell Imaging-From Pre-Clinical Validation to First in Man Testing. Int J Mol Sci (2020) 21(19):7274. doi: 10.3390/ijms21197274 PMC7582644 33019671

[86] Balhuizen A Massa S Mathijs I Turatsinze JV De Vos J Demine S. A Nanobody-Based Tracer Targeting DPP6 for Non-Invasive Imaging of Human Pancreatic Endocrine Cells. Sci Rep (2017) 7(1):15130. doi: 10.1038/s41598-017-15417-2 29123178 PMC5680294

[87] Demine S Garcia Ribeiro R Thevenet J Marselli L Marchetti P Pattou F. A Nanobody-Based Nuclear Imaging Tracer Targeting Dipeptidyl Peptidase 6 to Determine the Mass of Human Beta Cell Grafts in Mice. Diabetologia (2020) 63(4):825–36. doi: 10.1007/s00125-019-05068-5 31873789

[88] Kemter E Citro A Wolf-van Buerck L Qiu Y Bottcher A Policardi M. Transgenic Pigs Expressing Near Infrared Fluorescent Protein-a Novel Tool for Noninvasive Imaging of Islet Xenotransplants. Xenotransplantation (2021) 21:e12719. doi: 10.1111/xen.12719 34935207

